# Prevalence of Urinary Tract Infection in Children With Severe Acute Malnutrition Aged Between Six Months and Five Years and Their Antibiotic Sensitivity Pattern

**DOI:** 10.7759/cureus.45245

**Published:** 2023-09-14

**Authors:** Sweta Tiwari, Kesh Ram Meena, Rani Gera

**Affiliations:** 1 Paediatrics, Vardhman Mahavir Medical College and Safdarjung Hospital, Delhi, IND

**Keywords:** clinico-epidemiologic profile, child health care, antibiotic sensitivity pattern, urinary tract infection, severe acute malnutrition (sam)

## Abstract

Objective

This study was conducted to determine the prevalence of urinary tract infection in children with severe acute malnutrition (SAM) aged between six months and five years and to identify the causative organisms and their antibiotic sensitivity pattern.

Study design

This study was an observational cross-sectional study.

Setting and participants

The study was conducted in the Department of Paediatrics in a tertiary care hospital in India over a period of 18 months. A total of 140 children aged between six months and five years according to the World Health Organisation's criteria of severe acute malnutrition were included upon fulfillment of inclusion and exclusion criteria. Ultrasound of kidney, ureter, and bladder (USG-KUB) was also done to exclude children with any underlying anatomical anomaly.

Intervention

Detailed clinical examination was performed on each of the participants with emphasis on anthropometry. Relevant blood investigations were sent along with urine routine microscopy and culture sensitivity in all patients.

Results

The prevalence of urinary tract infection (UTI) in our study was 23.57%. The most common organism isolated was Escherichia coli found in 54.54% of cases, followed by Klebsiella in 24.24%. Other organisms isolated were Enterococcus in 12.12%, Pseudomonas aeruginosa 6.06% and Citrobacter in 3.03%. E. coli showed high sensitivity to imipenem (88.87%), meropenem (83.84%), nitrofurantoin (77.76%) and amikacin (72.23%). Overall these organisms showed good sensitivity to amikacin (60.06%), imipenem (66.6%), meropenem (63.63%) and nitrofurantoin (72.72%). Resistance to common antibiotics like ciprofloxacin, cefotaxime and cefuroxime was seen.

Conclusion

Children with malnutrition are at risk of UTI. Urine routine examination and urine culture should be performed in all these children before starting antibiotics. Selection of an antibiotic should be according to the local drug sensitivity data. These antibiotics should have good efficacy against gram-negative organisms.

## Introduction

Urinary tract infection (UTI) is an infection in any part of the urinary system, urethra, bladder, ureter or kidneys. Most UTIs are ascending infections and the bacteria arises from the faecal flora that colonize the perineum. Male preponderance is seen in infancy and female preponderance thereafter. The risk of having a UTI before the age of 14 years is found to be 3-10% in girls and 1-3% in boys. UTI in children can present with nonspecific symptoms like unexplained fever and failure to thrive or as a part of sepsis in a newborn [1,2]. Older children can have additional urinary symptoms like burning micturition, increased frequency of micturition and nocturnal enuresis. UTI can be a cause of pyelonephritis and chronic kidney disease and can also lead to invasive bacterial sepsis [3,4]. Hence early recognition and appropriate treatment are important.

Severe acute malnutrition (SAM) is a grave public health issue. According to the National Family Health Survey (NFHS-4) in India, among children under five years, 7.5% in rural and 7.4% in urban areas are severely wasted [5,6]. Malnutrition plays a major role in over one-third of all child deaths worldwide [7-9]. It is associated with immune deficiency especially cell-mediated immunity as well as a diminished inflammatory response that makes affected children more vulnerable to infections [10,11]. Malnutrition increases susceptibility to infections while infections aggravate malnutrition by decreasing appetite, inducing catabolism, and increasing demand for nutrients [12].

The response to infection is impaired or absent in children with malnutrition. So, the usual clinical features seen in UTI may not be present in these children. Hence a high suspicion is required in them. In malnutrition, urinary tract infection is very common which is 6-37% in literature reviews [1]. Not much information is available from developing countries, but some studies from Africa report it to be 9-11.4% [13,14] and few from India found it to be 15.2-22.3% [15-21]. Since there are limited studies available, hence this study was planned to determine the prevalence of urinary tract infection in children with severe acute malnutrition aged between six months and five years and to identify the causative organisms and their antibiotic sensitivity pattern.

## Materials and methods

This was a hospital-based observational cross-sectional study conducted in the Department of Paediatrics in one of the largest central government hospitals of India (Safdarjung Hospital, New Delhi) over a period of 18 months (from October 2019 to March 2021). The sample size was calculated using 22.4% prevalence from a previous study by Sharma et al. [15] with a 7% margin of error and a 5% level of significance, which came out to be 137 patients, so the total sample size taken was 140.

Children aged between six months and five years admitted in department of paediatrics of Safdarjung Hospital and meeting the World Health Organisation's (WHO) criteria for SAM were enrolled after taking informed consent from parents or guardian.

The WHO criteria for SAM are weight for height/length below -3 standard deviation of the median (using the WHO Growth Charts) or presence of visible severe wasting or presence of bipedal oedema of nutritional origin or mid-upper arm circumference less than 11.5 cm [22].

Children having congenital anomalies of the kidney and urinary tract, vesicoureteric reflux, nephrolithiasis or obstructive uropathy were excluded from the study with a prior ultrasound. We also excluded patients having any underlying renal disease, as these patients are already at a high risk for urinary tract infection. Patients on steroids for more than two weeks or those who had taken any antibiotics within one week prior to presentation were also excluded.

All subjects were evaluated on the basis of a preformed proforma including demographic details and required clinical history. Clinical examination was performed on each of the participants with emphasis on anthropometry (height, weight and mid-upper arm circumference). Blood investigations were sent which included complete blood count, C-reactive protein, kidney function test, serum electrolytes and blood culture sensitivity. Ultrasound of abdomen with kidney, ureters and bladder was done in all cases. Other specific investigations were done depending upon the history and clinical findings of the patient. Two urine samples were collected from each child before starting antibiotics for routine microscopy and culture sensitivity. In children more than two years of age, a clean-catch midstream specimen was taken after washing the genitalia with soap and water. In children less than two years of age or those in which midstream specimen could not be obtained such as the patients in shock, sample was collected using an appropriate size urinary catheter, after taking all aseptic precautions. These samples were sent to the microbiology laboratory within one hour of collection for routine microscopy, culture and antibiotic susceptibility tests. In case the child was admitted at night, the samples were stored in the refrigerator and sent the next following morning.

As soon as the samples were received in the microbiology laboratory, routine microscopy was performed. A drop of well mixed, un-centrifuged urine specimen was placed on a clean glass slide using a plastic dropper and it was covered with 22x22 mm cover slip avoiding bubble formation. After this the wet mount was examined under the microscope for the presence of pus cells and microorganisms.

Further, the urine sample was cultured on a sheep blood agar (SBA) and a MacConkey agar (MA) by the semi-quantitative method, wherein a one microlitre (μl) sterile nichrome loop (Himedia, Mumbai, India) was used to deposit the said amount of urine on a SBA and MA. The plates were then incubated for 18 - 24 hours at 35 - 37°C in an incubator. After overnight incubation, the number of colonies that had grown on the plates are counted manually and then the number was multiplied by 1000. This gave the total number of viable bacteria present in one ml undiluted urine and expressed as colony-forming unit (CFU)/ml of urine. Based on the growth characteristics on both the SBA and MA, the bacteria/bacterium was/were further identified by various biochemical tests and the antibiotic susceptibility test were performed using the Kirby Bauer’s disk diffusion method on a Mueller Hinton agar plate. The results for the bacterial identification and susceptibility pattern were read after an incubation at 35 - 37°C for 18 - 24 hours.

The confirmatory diagnosis of urinary tract infection was made only on the basis of urine culture report. Colony count of more than 10^5^ CFU/ml of a single species of bacteria in a midstream clean catch sample was considered as significant bacteriuria. For the samples collected by urethral catheterisation, a colony count of more than 5 × 10^4^ CFU/ml of single organism was considered significant [2].

All details and outcome were recorded in a predesigned proforma. 

Objective

The objective of this study was to determine the prevalence of urinary tract infection in children with severe acute malnutrition aged between six months and five years; and to identify the causative organisms and their antibiotic sensitivity pattern.

Statistical analysis 

The presentation of the Categorical variables was done in the form of number and percentage (%). On the other hand, the quantitative data were presented as the means ± standard deviation (SD) and as median with 25th and 75th percentiles (interquartile range). The association of the quantitative variables were analysed using independent t test and the association of the qualitative variables were analysed using Chi-Square test. If any cell had an expected value of less than five, then Fisher’s exact test was used. 

The data entry was done in an Excel spreadsheet (Microsoft, Redmond, WA, USA) and the final analysis was done using Statistical Package for Social Sciences (SPSS) software, version 21.0 (IBM Corp., Armonk, NY, USA). For statistical significance, p value of less than 0.05 was considered statistically significant. 

## Results

In this observational cross-sectional study 142 children were initially enrolled after proper history and clinical examination; two of them were excluded due to abnormal renal ultrasound findings. One hundred forty children fulfilling the inclusion and exclusion criteria were further investigated.

In our study most of the children were in the age group between six to 12 months (35% patients) followed by 13 to 24 months (29.29%), 25 to 36 months (15.00%), 37 to 48 months (12.14%) and 49 to 60 months (8.57%). Mean value of age (months) of study subjects was 24.47 ± 16.5 with median (25th-75th percentile) of 21 (11.04-36). The study population had 76 females (54.29%) and 64 males (45.71%) (Table 1).

**Table 1:** Distribution of study population according to age and gender.

Demographic characteristics	Frequency	Percentage
Age(months)		
6-12 months	49	35.00%
13-24 months	41	29.29%
25-36 months	21	15.00%
37-48 months	17	12.14%
49-60 months	12	8.57%
Mean ± SD	24.47 ± 16.5	
Median (25th-75th percentile)	21(11.04-36)	
Range	6-60	
Gender		
Female	76	54.29%
Male	64	45.71%

Mean value of weight (kg), height (cm) and mid-upper arm circumference (cm) of study subjects was 7.54 ± 2.11, 81.52 ± 12.54 and 10.27 ± 0.95 respectively. A total of 127 (90.7%) patients out of 140 patients had weight for height less than -3 standard deviation (SD). Mid-upper arm circumference less than 11.5 cm was seen in 122 (87.14%) patients. Edema was present in only four (2.85%) patients and visible severe wasting was seen in 63 (45%) patients. No significant association of UTI was seen with any of the above parameters (Table 2).

**Table 2:** Association of anthropometric parameters with urinary tract infection (UTI) † Fisher's exact test, ‡ Chi square test

Nutritional status	UTI absent(n=107)	UTI present(n=33)	Total	P value
Weight for height	97 (76.38%)	30 (23.62%)	127 (100%)	1^†^
Mid upper arm circumference(<11.5cm)	95 (77.87%)	27 (22.13%)	122 (100.00%)	0.296^‡^
Edema	2 (50%)	2 (50%)	4 (100%)	0.236^†^
Visible severe wasting	48 (76.19%)	15 (23.8%)	63 (100%)	0.1^†^

**Figure 1:**
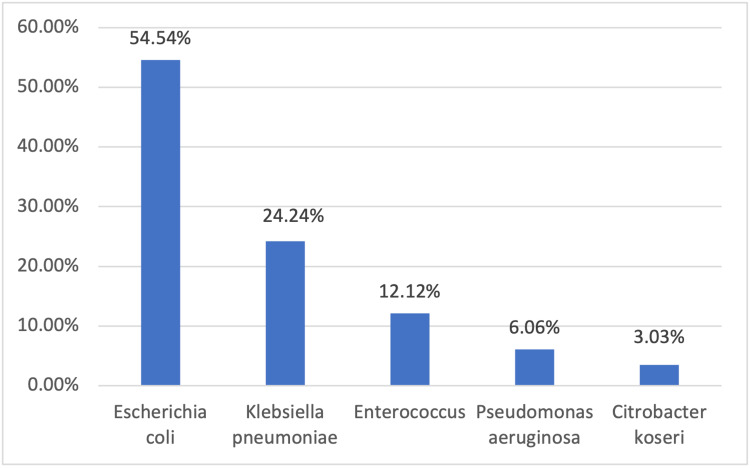
Distribution according to type of organism grown

In the present study, the majority (76.43%) of patients did not have any growth on urine culture. Out of 140 patients, urine culture was positive in 22 (23.37%). UTI was more common in females, present in 22 out of 76 females (28.95%) compared to 11 out of 64 males (17.19%) (p value-0.102). The most common organism was E. coli, isolated in 18 (54.54%) children, followed by Klebsiella pneumoniae in eight (24.24%), Enterococcus species in four (12.12%), Pseudomonas aeruginosa in two (6.06%) and Citrobacter koseri in one out of 33 children (3.03%) (Figure 1).

E. coli showed high sensitivity to imipenem (88.87%), meropenem (83.84%), nitrofurantoin (77.76%), amikacin (72.23%) and comparatively lower sensitivity was seen for cotrimoxazole (44.45%), amoxycillin-clavulanic acid (44.45%) and ciprofloxacin (22.23%). Other organisms and their sensitivity pattern are shown in Table 3.

**Table 3:** Organism and sensitivity pattern

Drugs	E. coli(18)	Klebsiella(8)	Pseudomonas(2)	Enterococcus(4)	Citrobacter(1)	Total(33)
Ciprofloxacin	4(22.23%)	0(0%)	0(0%)	1(25%)	0(0%)	4
Amikacin	13(72.23%)	6(75%)	1(50%)	0(0%)	1(100%)	20
Amoxyclav	8(44.45%)	3(37.5%)	-	-	0(0%)	11
Cotrimaxazole	8(44.45%)	2(25%)	-	-	0(0%)	10
Meropenem	15(83.84%)	7(87.5%)	1(50%)	-	1(100%)	21
Imipenem	16(88.87%)	6(75%)	1(50%)	-	1(100%)	22
Nitrofurantoin	14(77.76%)	4(50%)	-	4(100%)	1(100%)	24
Cefuroxime	0(0%)	0(0%)	-	-	-	0
Cefotaxime	0(0%)	0(0%)	-	-	0(0%)	0
Vancomycin	-	-	-	4(100%)	-	4
linezolid	-	-	-	4(100%)	-	4

In urine microscopy, the majority of children had a pus cell count of less than five (88.57%), followed by five to 10 pus cells in 8.57% of cases. Pus cells were more than 10 in only four out of 140 patients (2.86%). The proportion of patients with UTI was proportionately higher in the group having more numbers of pus cells. Among children having five to 10 pus cells in urine, 66.67% had UTI. This proportion was even higher for the group having more than 10 pus cells as 75% of these children had UTI. This was higher as compared to only 17.74% of children with less than five pus cells had UTI. This association was statistically significant with a p value of less than 0.0001 (Table 4).

**Table 4:** Association of pus cells in urine with urinary tract infection (UTI)

Pus cells	UTI absent(n=107)	UTI present(n=33)	Total	P value
<5	102 (82.26%)	22 (17.74%)	124 (100%)	<.0001^†^
5-10	4 (33.33%)	8 (66.67%)	12 (100%)	
>10	1 (25%)	3 (75%)	4 (100%)	
Total	107 (76.43%)	33 (23.57%)	140 (100%)	

In the majority (59.29%) of patients, presenting complaint was fever followed by loose stool (27.14%), cough/fast breathing (13.57%), vomiting (10.71%), not gaining weight (7.14%), lethargy/decreased oral acceptance (5.71%), pallor (12.85), seizure (1.43%) and pain abdomen (1.43%). Burning micturition as a presenting complaint was seen in only one out of 140 patients (0.71%). However it was found to have 100% association with UTI in our study. Among presenting complaints only presence of loose stool was found to have a statistically significant association with UTI with a p value of less than 0.0239 (Table 5).

**Table 5:** Association of presenting complaints with urinary tract infection (UTI) † Fisher's exact test, ‡ Chi square test

Presenting complaints	UTI absent(n=107)	UTI present(n=33)	Total	P value
Fever				
Negative	48 (84.21%)	9 (15.79%)	57 (100%)	0.072^‡^
Positive	59 (71.08%)	24 (28.92%)	83 (100%)	
Cough/fast breathing				
Negative	93 (76.86%)	28 (23.14%)	121 (100%)	0.762^‡^
Positive	14 (73.68%)	5 (26.32%)	19 (100%)	
Not gaining weight /failure to thrive				
Negative	100 (76.92%)	30 (23.08%)	130 (100%)	0.7^†^
Positive	7 (70%)	3 (30%)	10 (100%)	
Pallor				
Negative	94(77.04%)	28(22.95%)	122 (100%)	0.652^‡^
Positive	13(72.22%)	5(27.77%)	18(100%)	
Vomiting				
Negative	97(77.6%)	28 (22.4%)	125 (100%)	0.346^‡^
Positive	10(66.67%)	5(33.34%%)	15 (100%)	
Loose stool				
Negative	83(81.38%)	19(18.62%%)	102 (100%)	0.0239^‡^
Positive	24(63.15%)	14 (36.84%)	38 (100%)	
Seizure				
Negative	107 (77.54%)	31 (22.46%)	138 (100%)	0.054^†^
Positive	0 (0%)	2 (100%)	2 (100%)	
Pain abdomen				
Negative	106 (76.81%)	32 (23.19%)	138 (100%)	0.417^†^
Positive	1 (50%)	1 (50%)	2 (100%)	
Lethargy/decreased oral acceptance				
Negative	101 (76.52%)	31 (23.48%)	132 (100%)	1^†^
Positive	6 (75%)	2 (25%)	8 (100%)	
Burning micturition				
Negative	107 (76.97%)	32 (23.02%)	139 (100%)	0.236^†^
Positive	0(0%)	1(100%)	1 (100%)	

## Discussion

The overall prevalence of UTI in children with SAM in our study was 23.57% (33 out of 140 children). Most of the study population was in the age group of six to 12 months (35%) and most cases of UTI were seen in the age group of 13-24 months (42.42% of total UTI cases). In our study 76 (54.29%) patients were females and 64 (45.71%) patients were males. UTI was found to be more common in females as seen in 22 out of 54 females (28.95%) as compared to 11 out of 53 males (17.19%). However, this association was not statistically significant (p value=0.102). This was similar to previous studies [18,20,21] where a significant association of UTI with female gender was seen. The female predominance of UTI in malnourished children is in accordance with female preponderance of UTI in general. This may be due to shorter urethra seen in females leading to more chances of ascending infections.

In our study fever was the most common presenting complaint (59.29%) followed by loose stool (27.14%), cough/fast breathing (13.57%), pallor (12.85%), vomiting (10.71%), not gaining weight (7.14%), lethargy/decreased oral acceptance (5.71%), seizure (1.43%) and pain abdomen (1.43%). The presenting complaint was burning micturition in only one out of 140 patients (0.71%). Fever was present as the most common presenting complaint in other studies by Sharma et al. [15] (84.7%), Kumar et al. [19] (59.3%) and Dangayach et al. [20] (33.84%), whereas respiratory symptoms were found to be most common in studies by Dey et al. [21] and Banapurmath et al. [17]. Among all these clinical presentations studied, only loose stool was found to have a statistically significant association with UTI in our study (p value 0.0239). It was similar to the study done by Bagga et al. [3] where UTI was present in 23.3% of children with diarrhoea compared to 10.1% in those without diarrhoea (P value<0.05). The high incidence of UTI in patients with diarrhoea may be related to the increased colonisation of periurethral flora in these patients. Urinary symptoms were not a common finding as seen in only one (0.71%) patient. However, it was found to have a 100% association with UTI in our study as well as other studies [15,20].

We found the proportion of patients having UTI was significantly higher in the group having more than five pus cells, i.e., five to 10 pus cells (66.67%), >10 pus cells (75%) as compared to less than five pus cells (17.74%). This association was found to be statistically significant in our study with a p value <.0001. Similar significant association was found in most other studies [3,19,20]. On the contrary, a study by Ibrahim et al. [18] and Rabasa et al. [16] found poor leucocyte response in children with malnutrition compared to control. So, pyuria cannot be used as a criterion to diagnose UTI as it can be present without bacteriuria in various conditions like infections elsewhere in the body or glomerulonephritis. But presence of pyuria should increase the suspicion of UTI as seen in our study and various other studies as well.

The prevalence of UTI in our study was 23.57% (33 out of 140 patients were urine culture positive). It was comparable to some other studies [15,20].

In our study, the most common organism isolated was E. coli, present in 18 samples (54.54%). Followed by Klebsiella in eight (24.24%), Enterococcus in four (12.12%) and Pseudomonas aeruginosa in two (6.06%) and Citrobacter koseri in only one out of 33 patients (3.03%). E. coli showed high sensitivity to imipenem (88.87%), meropenem (83.84%), nitrofurantoin (77.76%), and amikacin (72.23%). Comparatively lower sensitivity was seen for cotrimoxazole (44.45%), amoxycillin-clavulanic acid (44.45%) and ciprofloxacin (22.23%). Klebsiella also showed high sensitivity to imipenem (75%), meropenem (87.5%) and amikacin (75%) and comparatively lower sensitivity for nitrofurantoin (50%), cotrimoxazole (25%), amoxycillin-clavulanic acid (37.5%) and all were resistant to ciprofloxacin. None of the isolates of E. coli or Klebsiella showed 100% sensitivity to any of the antibiotics. Pseudomonas showed 50% sensitivity to imipenem, meropenem and amikacin. All the Enterococci were sensitive to vancomycin, linezolid and nitrofurantoin (100%) and none were sensitive to amikacin. Citrobacter was 100% sensitive to imipenem, meropenem, nitrofurantoin and amikacin and resistant to cotrimoxazole and amoxycillin-clavulanic acid. None of the isolates were sensitive to cefotaxime or cefuroxime. In contrast, previous studies showed good sensitivity to amikacin and ciprofloxacin. In study by Kumar et al. [19] organisms were 100% sensitive to amikacin, 81.4% to ciprofloxacin and 7% to cefotaxime. Bagga et al. [3] found good sensitivity to co-trimoxazole, amoxicillin, ciprofloxacin and ceftriaxone. In study by Ibrahim et al. [18] 100% sensitivity was seen to gentamycin and ciprofloxacin. In study by Sharma et al. [15] and Dangayach et al. [20], E. coli showed 90- 100% sensitivity to imipenem, amikacin, nitrofurantoin and gentamicin. In our study organisms were found to be more resistant to various antibiotics in comparison to previous studies. So we recommend antibiotics be selected based on the data available on sensitivity pattern of that hospital or region.

Strength of our study was that it had a good sample size compared to most of the previously done studies. Ultrasound was performed in all patients to exclude patients with structural defects in kidneys or urinary tract. Demographic characteristics of the population as well as association of UTI with clinical presentation was also studied. The main limitation of this study was that it was a hospital-based study that included only admitted patients, so it failed to estimate the prevalence of UTI in asymptomatic SAM patients and in general population. Controls were also not enrolled.

## Conclusions

Our study showed a high prevalence of UTI in children with severe acute malnutrition. The most common organisms isolated were gram-negative organisms, E. coli being the most common. Most of them showed good sensitivity to antibiotics like amikacin and nitrofurantoin and resistance to cefotaxime, cefuroxime and ciprofloxacin. Presence of pus cells in urine routine microscopy was found to be positively associated with UTI however a positive urine culture is always required to make the diagnosis of UTI. In our study presence of diarrhoea was found to be positively associated with UTI. It can be due to the increased colonisation of periurethral flora in these children. Among presenting complaints fever was most common. Urinary complaints were seen in less than 1% of cases in our study, so a high degree of suspicion is required in these children for UTI.

From this study we can conclude that children with malnutrition are at risk of UTI. Urine routine examination and urine culture should be performed in all these children before starting antibiotics. Selection of empirical antibiotic should be according to the local sensitivity data. If such data is not available, antibiotics having good efficacy against gram-negative organisms should be started till the time culture reports are collected. 
